# Attention Bias and Recognition of Sexual Images

**DOI:** 10.3389/fpsyg.2020.556071

**Published:** 2020-11-02

**Authors:** Ondřej Novák, Klára Bártová, Václav Vagenknecht, Kateřina Klapilová

**Affiliations:** ^1^Faculty of Humanities, Charles University, Prague, Czechia; ^2^Laboratory of Evolutionary Sexology and Psychopathology, National Institute of Mental Health, Klecany, Czechia

**Keywords:** attention, visuospatial attention, sexual, erotic, dot probe, recognition, pictures, images

## Abstract

Attention to sexual stimuli is necessary for the development of sexual response, yet while there is some evidence of attention bias in favor of sexual stimuli, the direction and magnitude of the effect remain unknown. A high-powered sample of 113 participants was tested using the dot-probe task (DPT) and picture recognition task (PRT) to measure visuospatial attention to erotic images. Participants showed no attention bias in the DPT (*r*_B_ = 0.201, *p* = 0.064) but were significantly better at recognizing erotic rather than neutral or training pictures (*d* = 1.445 and 1.461, respectively, both *p* < 0.001). This indicates that spatial attention bias to sexual pictures is small, negligible, possibly even non-existent, or else the DPT is not a reliable tool to assess it. Results of the PRT, on the other hand, show that sexual stimuli are prioritized in memory and this should be explored in future research.

## Introduction

Do sexual stimuli capture attention? In our culture it is assumed they do. This belief is reflected, for instance, in the frequent use of sexual content in advertising (Reichert et al., 1999; Reichert and Carpenter, 2003; Hennink-Kaminski and Reichert, 2011; Cummins et al., 2020) despite its questionable effect (Reichert and Alvaro, 2001; Cummins et al., 2020).

It has been demonstrated that attentional processes play a central role in sexual arousal (Barlow, 1986; de Jong, 2009) and it is believed that they are processed much like other evolutionarily meaningful stimuli (Spiering and Everaerd, 2007). Nearly all theoretical concepts which operate with attention and sexual stimuli assume some form of attention bias (for an evolutionary psychology-informed account, see Bailey et al., 1994). Surprisingly, though, only a handful of studies had so far investigated the actual patterns of attention toward sexual stimuli and even less studies employed cognitive tasks to do so.

In the context of sexual stimuli, two forms of attention are relevant: *cognitive attention* which takes the form of working memory capacity assigned to cognitive processing of a stimulus, and *visuospatial attention* in the form of directing one’s gaze to a stimulus (Imhoff et al., 2019). Cognitive attention to sexual stimuli has been reliably established with respect to the phenomenon of sexual content-induced delay (SCID), which describes a general slowing in cognitive tasks when sexual stimulus is present (Geer and Bellard, 1996). Most studies which use common paradigms for assessing attention – such as the modified Stroop task, parallel decision task, inclusive decision task, or visual search task (for a review, see Jiang and Vartanian, 2018) – in fact measure cognitive attention. Studies on visuospatial attention, on the other hand, are scarce and their results are far from clear-cut.

Two general paradigms are used to study visuospatial attention: one uses an eye-tracking device, the other works with the dot-probe task (DPT). In the eye-tracking paradigm, researchers assess early attentional processing (initial orienting) by measuring the position of the first (or second, see Rupp and Wallen, 2007; Fromberger et al., 2012) fixation while presenting the subject with two concurrent stimuli, e.g., a neutral and a sexual one. Regrettably, none of the eye-tracking studies reviewed below uses such clear design. Most existing studies use first fixation as an indicator of preferred sexual targets in various populations (e.g., heterosexual males: Dagnino et al., 2012; Fromberger et al., 2012; males with a pedophilic preference: Fromberger et al., 2013; and bisexual males and females: Morandini et al., 2020), thus effectively measuring attention bias with respect to different categories of sexual stimuli. In other studies, stimuli are presented in a series (e.g., Rupp and Wallen, 2007; Ganesan et al., 2020), or what is measured are late attentional processing indexes, such as viewing time and the total number of fixations (e.g., Lykins et al., 2006). Still, while eye-tracking studies show that sexually relevant stimuli are visually attended to, they fail to distinguish between cognitive and visuospatial attention bias (Imhoff et al., 2019).

The dot-probe task (DPT; also known as serial probe task, letter probe task, attentional deployment task, or visual probe task) is one of the most commonly employed tools used to assess visuospatial attention bias. Introduced in the context of research of attention to threat-related words in a clinically anxious population (MacLeod et al., 1986), the DPT was developed to measure early automatic allocation of spatial attention. It is based on the simple assumption that people respond faster to probes presented in the attended rather than unattended region of visual display (Posner et al., 1980; Navon and Margalit, 1983). In a typical setup, the subject is presented with pairs of stimuli (e.g., sexual and neutral pictures or words), which are then replaced by a probe that appears in the location of one of the stimuli. Subjects are asked to localize or identify the probe as quickly as possible. Faster response to a probe replacing a sexual stimulus would suggest that attention was directed to this stimulus (*vigilance*), while a slower response would indicate that attention was directed away from it (*avoidance*). The difference in mean response times between trials where the probe replaced a neutral picture (neutral target trials) and those where the probe replaced a sexual picture (sex target trials) amounts to attention bias index, extreme scores of which are thought to reflect attention bias (MacLeod et al., 1986). One of the advantages of the dot-probe paradigm is its ability to distinguish between attentional vigilance and avoidance. Moreover, its ecological validity can be increased by using pictures instead of words (Jiang and Vartanian, 2018). It can be also used to examine the progress of attention by varying the duration of exposure of stimulus pairs. While there is a considerable variation in stimulus exposure time used in various studies – it can vary from 100 ms (Cooper and Langton, 2006) to 2000 ms (Pottage and Schaefer, 2012) – most studies present the stimuli for 500 ms (Mogg et al., 2004; Prause et al., 2008; Kagerer et al., 2014). The main advantage of the dot-probe paradigm lies in its ability to distinguish between cognitive and visuospatial attention by using different combinations of stimuli pairs in trial conditions. The SCID effect will cause slightly longer response times in every trial where sexual stimulus is present. That is why researchers find consistently slower responses in trials with sexual content than in trials with neutral content in the modified Stroop task or parallel decision task. The dot-probe paradigm keeps the SCID effect constant because it has sexual content in every trial, with only the position of the probe changing. Any difference in the speed or accuracy of response between trials can therefore be attributed to potential bias in visuospatial attention (Imhoff et al., 2019). When researchers want to observe the SCID effect, they can simply add a trial condition consisting of two neutral stimuli.

Although the importance of attention in sexual functioning is beyond doubt, since it determines whether a sexual response will occur (Barlow, 1986), there is no clear consensus on how exactly people attend to sexual stimuli besides the SCID effect. Recently, Strahler et al. (2019) published a meta-analysis on attention bias toward and distractibility by sexual cues. Combining the results of 2933 participants in 32 studies (including eight studies that used the DPT) they found a medium-sized effect of *g*_*z*_ = 0.49, 95% *CI* [0.37, 0.61] for attention to sexual cues in general and an effect size of *g*_*z*_ = 0.34, 95% *CI* [0.17, 0.50] for the DPT (and letter probe) in particular. Nevertheless, due to differences in the methodologies used, the attention bias as measured by this meta-analysis combined several – and sometimes opposite – effects. To provide a more detailed analysis, we offer in the following a full review of studies which focused on visuospatial attention to sexual pictures.

Beard and Amir (2010) employed a modified dot-probe paradigm with sexual and neutral words to compare attention bias in women with low and high sexual functioning. In the sexually low-functioning group, they indeed found attention bias toward sexual words (η^2^ = 0.106), but in the other group, they found no such effect. In could be argued, though, that the power of verbal stimulus rests in its semantic associations and not in specific perceptual features (Oosterwijk et al., 2017), which is why it may be preferable to use visual stimuli, such as pictures or photographs (Jiang and Vartanian, 2018).

The first authors to measure visuospatial attention to sexual stimuli using a picture version of the DPT were Prause et al. (2008), who focused on the relation between attentional and emotional response to sexual stimuli and participants’ sexual desire. Although the authors did not report exact values, their general analysis shows that participants displayed slower reactions to probes in sex target trials than to probes in neutral target trials (*η^2^_*p*_* = 0.46). Prause and colleagues also found an unexpected negative relationship between attention bias and a broadly defined latent factor of sexual desire: participants with higher levels of sexual desire were slower to detect probes in sex target trials (*η^2^_*p*_* = 0.16). The effect was rather robust and affected three major domains of sexual desire (desire for autoerotic sexual activity, desire for sexual activity with a partner, and overall propensity for sexual excitation).

A similar pattern of response times was found in a study which compared attention bias for sexual pictures in women with and without hypoactive sexual desire disorder (Brauer et al., 2012). As in the previous study, participants were in general slower at detecting probes in sex target trials compared to neutral target trials (η^2^*_*p*_* = 0.045 for the hypoactive sexual desire disorder group and η^2^*_*p*_* = 0.079 for sexually functional group). Given that sexual stimuli are considered highly salient (Spiering and Everaerd, 2007) and as such should attract attention, thus allowing for faster reactions after replacement by a probe, these results are somewhat counterintuitive.

Several later studies used a picture DPT in slightly different contexts. Doornwaard et al. (2014) investigated how short erotic film clips affect the performance of the task. In this study, participants responded faster in sex target trials than in neutral target trials (η^2^*_*p*_* = 0.07) but viewing of erotic clips had no effect on their performance. Kagerer et al. (2014) published similar results with a slightly smaller effect size (η^2^ = 0.027). Another study compared the attention bias index in individuals with and without compulsive sexual behavior disorder (Mechelmans et al., 2014). Both the clinical group and controls had positive and non-zero attention bias toward explicit sexual stimuli, which manifested itself in faster responses in sex target trials than in neutral target trials. Regrettably, the authors provided no values or effect sizes besides *p*-values. Employing the mTurk web platform, Seehuus (2015) compared the effect of different exposition times in the DPT. He found a significant positive attention bias in sex target trials for presentation times of 50, 500, and 1250 milliseconds (η^2^ = 0.129, 0.021, and 0.008, respectively). Following up with two experiments, Snowden et al. (2016) focused on gender–specific differences in target stimuli. In the first experiment, rather than pair a sexual picture with a neutral one, they used pictures containing a nude male and a nude female. Their study also employed shorter exposure times (200 ms). The results showed that heterosexual men reacted faster when probe replaced a picture of a female (η^2^ = 0.57), while in heterosexual women, no such difference was found. In their second experiment, they added neutral pictures. In line with the previous experiment, both men and women reacted faster to probes that followed pictures of females in both male–female (η^2^ = 0.172 and 0.075, respectively) and female–neutral trial (η^2^ = 0.265 and 0.075, respectively), while in male–neutral trials, there was no difference in reaction times. Visual processing of sexual stimuli tends to be strongly influenced by sex (Dawson and Chivers, 2016) but while the cultural effects of sexual responding are more accentuated in women (Ganesan et al., 2020), men’s reactions tend to be consistent. For example, males are strongly motivated by sexual stimuli (Dewitte, 2016), show greater memory bias for erotic elements in stories (Geer and McGlone, 1990), and display a more profound SCID effect (Conaglen and Evans, 2006). But this is the only study that found any difference between men and women in the dot-probe paradigm.

Such mixed evidence may be the result of low reliability of the dot-probe paradigm as such (Schmukle, 2005; Staugaard, 2009). After all, an unreliable tool measures only error variance, thus leading to inconsistent results across studies (Schmukle, 2005). Snowden et al. (2016) suggest that the relatively low reliability of DPT studies could be linked to a relatively small or perhaps even non-existent effect they are trying to detect. Another source of this inconsistency could be publication bias, a phenomenon that is widespread both in cognitive sciences in general (Ioannidis et al., 2008) and in research on attention to sexual stimuli research in particular (Strahler et al., 2019). Complete absence of non-significant results in published studies seems to support this explanation and in conjunction with high heterogeneity of results in reviewed studies (Strahler et al., 2019), we thus see a clear need of more high-powered studies.

In the light of concerns about DPT’s ability to assess early automatic allocation of spatial attention (see, e.g., Cooper and Langton, 2006), it is desirable to employ some kind of validation measure (e.g., Miller and Fillmore, 2010). When searching for an appropriate instrument to validate the DPT results, we considered the following observations made in previous studies: (1) subjects tend to spend more time looking at highly valued pictures, (2) they recognize pictures they looked at longer irrespectively of picture evaluation, and (3) subjects form no memory of pictures viewed only peripherally (Loftus, 1972). Based on the logic of the DPT, MacLeod et al. (1986) suggested that sexual stimulus should capture subject’s attention as soon as it appears on the screen and remain its focus until the probe is revealed. If attention bias toward sexual pictures is present, attention should be largely directed at sexual stimuli. And since attention to a stimulus is crucial for a successful creation of memory of it (Bush and Geer, 2001; Pottage and Schaefer, 2012), subjects should be later able to recognize significantly more sexual than neutral pictures. Following this reasoning, we employed in our study a simple picture recognition task (PRT). Previously, such recognition tasks were used with sexual stimuli for assessing SCID (Conaglen and Evans, 2006) or in the context of advertisement research (for a review, see Wirtz et al., 2017). To the best of our knowledge, however, we are the first to use a PRT as an indicator of visuospatial attention to sexual stimuli.

While most of the reviewed studies demonstrated that the presence of sexual pictures had some effect on attention to probes, the magnitude and direction of measured attention bias was highly inconsistent. Some studies reported faster reactions when a probe replaced a sexual picture (Doornwaard et al., 2014; Kagerer et al., 2014; Mechelmans et al., 2014; Seehuus, 2015; Snowden et al., 2016), while others observed slower responses in such cases (Prause et al., 2008; Brauer et al., 2012). We decided to contribute to this body of research with a highly powered study to see whether we can find support for either of the observations. Although only one of the reviewed studies reported sex differences in the visuospatial attention bias, we decided to test for sex differences in our sample to be consistent with the previous literature on sexual responding. In order to explore further the relationship between the visuospatial attention bias and sexual desire, we employ a revised Sociosexual Orientation Inventory (SOI–R; Penke and Asendorpf, 2008). While Prause et al. (2008) measured mainly dyadic sexual desire, SOI-R assesses a more general desire for sexual variability, which might better reflect previous findings when using stimuli that feature more than one sexual target. We use Beck Depressive Inventory (BDI-II; Ptáček et al., 2016) to further characterize the sample (e.g., like Beard and Amir, 2010) and to control for depressive symptoms which could negatively affect sexual desire levels in general (Frohlich and Meston, 2002) and therefore also any attention directed at sexual stimuli.

The aim of the present study is to contribute to our knowledge of the magnitude and direction of the assumed attention bias toward sexual stimuli in a nonclinical population as measured by the DPT. Moreover, we are the first to use the PRT to validate DPT results while controlling for the effects of sex, depressive symptoms, and sociosexuality.

## Materials and Methods

### Participants

An *a priori* power analysis was conducted using G^∗^Power 3.1.9.2 (Faul et al., 2007) to test the difference between two dependent group means using a two-tailed *t*-test, a medium effect size *d* = 0.34 (effect size estimate for DPT with sexual stimuli given by Strahler et al., 2019), and an alpha of 0.05. The results showed that a sample of 93 participants was required to achieve a power of 0.90.

A total of 119 Czech students were recruited at the Charles University campus. Data from six participants (four women, two men) were excluded from analyses due to high error rate (20% or more during the DPT), leaving 113 participants (*M*_age_ = 22.00, *SD*_age_ = 4.47, and 60.2% females). This number was substantially higher than the desired sample size. All participants received a remuneration of 100 CZK (app. 4 EUR) for participation. Levels of depressive symptoms in our sample (*M* = 11.848, *SD* = 9.254) were comparable to those of general healthy Czech population under thirty (*M* = 10.73, *SD* = 11.53; Ptáček et al., 2016).

### Measures

The DPT design was initially partially adapted from Prause et al. (2008) but during pilot testing, most participants found it too easy. To increase the difficulty, we employed a modified version of the DPT where participants were asked to respond to probe direction rather than merely its position. Both DPT and PRT were run using E-Prime software package 2.0 (Schneider et al., 2002).

#### The Modified Dot-Probe Task

**FIGURE 1:**
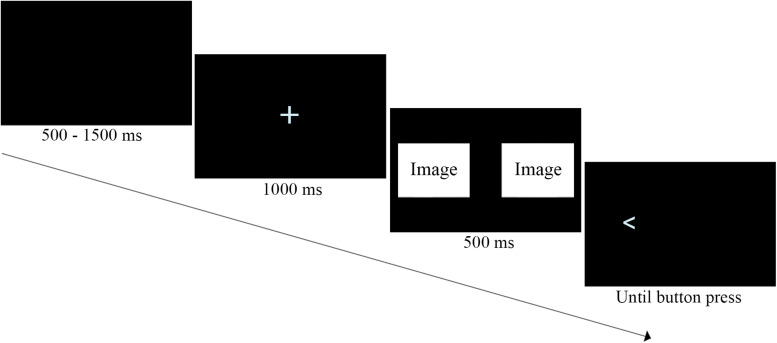
Modified dot-probe task trial.

In the DPT, each trial started with an intertrial black screen displayed for 500, 750, 1000, or 1500 milliseconds followed by a fixation cross (1000 ms). Next, laterally randomized sexual and neutral pictures were simultaneously presented on the screen for 500 ms. Then the pictures disappeared and one of them was replaced by a probe in the form of an arrowhead pointing left or right, which stayed on the screen until a participant responded (see Figure 1). Participants were instructed to press quickly the key assigned to probe’s direction (Q for arrow pointing left, P for arrow pointing right). Lateral position of the probe, which picture category it replaced, and which direction it indicated were all randomized. The DPT started with 20 training trials and continued with two blocks of 50 experimental trials, whereby each block used identical but randomly paired pictures.

#### Picture Recognition Task

The PRT followed the yes/no task paradigm. Participants were presented with a series of 365 pictures, where signal trials contained pictures previously encountered in the DPT (50 sexual, 50 neutral, and 40 training pictures) and noise trials were taken from pictures not selected for any experimental condition in the pilot study (see section “Materials”). There were 255 distractor pictures, which corresponded to 62% of presented stimuli. By pressing assigned keys, participants were instructed to indicate whether they saw the picture previously during the DPT (P for “seen”) or not (Q for “not seen”). Participants went through the PRT at their own pace because every picture remained on the screen until a response was recorded.

#### Questionnaires

All participants completed a short battery of questionnaires consisting of the Screening Questionnaire for Psychiatric Disorders, BDI–II (Beck et al., 1996), SOI-R (Penke and Asendorpf, 2008), and Emotion Regulation Questionnaire (ERQ; Gross and John, 2003). Back-translated and standardized Czech version of BDI-II (Ptáček et al., 2016) and back-translated versions of SOI-R and ERQ questionnaires were used. ERQ values were collected for different study and will be reported elsewhere.

### Materials

All pictures used in the study were taken from standardized datasets, namely IAPS (Lang et al., 1997) and NAPS (Marchewka et al., 2014). A total of 365 pictures depicting male–female couples, males, and females were pre-rated by 280 online raters (*M*_age_ = 28.94, *SD*_age_ = 6.23, 51% females) for the sexual/non-sexual content. Raters viewed a series of 80 pictures in a random order. They were asked to categorize each picture to sexual or non-sexual category (binary choice). For each picture, we obtained on average 48 ratings, from which we computed the percentual rating for sexual category (see Supplementary Material). Based on these ratings, we selected 57 sexual (sexual rating ranging from 63.9% to 100% with the mean of 93.11%) and 97 neutral pictures (sexual rating ranging from 0% to 17.6% with the mean of 2.78%). 43 sexual pictures depicted naked heterosexual pairs engaging in sexual activities, while 14 sexual pictures depicted nudity (seven showed a naked man and seven a naked woman). These latter pictures were used in the female and male version of the task, respectively. For each of the 50 sexual pictures, there was a neutral picture matched for content. In other words, these neutral pictures depicted mainly clothed heterosexual pairs, single women, or single men. Another 40 more neutral pictures depicting clothed men (one man per picture) were used for the training phase of the DPT. The rest of the 365 original pictures was used as distractors for the PRT (sexual rating ranging from 0 to 93.5% with a mean of 30.15%; see Supplementary Material).

### Procedure

Students were addressed by an assistant at the university campus and offered participation in the study. Upon agreeing to participate, they received further information to read, their eventual questions were answered, and all participants signed the informed consent form. At the outset, each participant completed the Screening Questionnaire, BDI–II, SOI–R, and ERQ. Next, each participant was seated in front of a personal computer and the DPT and PRT were administered. Finally, each participant was debriefed and before leaving received remuneration. The entire procedure took app. 30 min.

### Data Analysis

Data analyses were performed using R (R Core Team, 2019) and JASP (JASP Team, 2019). DPT reaction times (RT) were trimmed by incorrect responses (0.67% of all data) and values 4 *SD* above and below group mean (2.65% of all data). Descriptive statistics are presented in Table 1. Due to technical issues, data were lost from 8 participants for BDI–II and 11 participants for SOI–R.

**TABLE 1:** Dot-probe task: Mean RT (ms), median, and *SD*.

	**All (*N* = 113)**			**Men (*n* = 45)**			**Women (*n* = 68)**		
	**Mean**	**Median**	***SD***	**Mean**	**Median**	***SD***	**Mean**	**Median**	***SD***
All trials	628.242	613.950	128.926	601.969	603.768	106.153	645.629	621.594	140.056
Sex target	624.914	611.059	131.810	597.889	608.260	104.692	642.799	612.551	145.016
Neutral target	631.654	615.408	127.603	606.063	597.306	109.213	648.590	636.190	136.582
AB	3.370	2.495	14.607	4.087	2.495	13.169	2.895	2.340	15.563

*AB, Attention bias index.*

Attention bias index was computed for each participant by subtracting the mean reaction time in sex target trials from the mean reaction time in neutral target trials. Positive values indicate vigilance to sexual pictures, negative values indicate avoidance of sexual pictures.

Because of normality violations assessed by Shapiro–Wilk test (*p* < 0.001), we calculated differences in reaction times between neutral target trials and sexual target trial using two-tailed Wilcoxon signed-rank tests. Two-tailed Mann–Whitney *U* test was used to calculate the differences in attention bias index between men and women. Because of concerns regarding low reliability of the dot-probe paradigm (Schmukle, 2005; Staugaard, 2009), split-half reliability estimates were calculated by correlating the first half of the trials with the second half.

The relationship between attention bias index and sum scores of the questionnaires was tested using Spearman’s rank correlation coefficient.

The PRT was evaluated using signal detection theory. We also calculated hit rates for each picture category (sexual, neutral, and training) and false alarm rates. Sensitivity index (*d’*) and response bias index (*c*) were calculated for sexual, neutral, and training conditions for each participant (Stanislaw and Todorov, 1999). The false alarm rates were low (*M* = 12.2%, *SD* = 12.2%). Mixed ANOVA was performed for hit rates, sensitivity index *d’*, and response bias index *c* with one within-subject factor (Category: sexual, neutral, and training) and one between-subject factor (Sex: men, women).

For all tests, we set the alpha level of statistical significance 0.05 and calculated effect sizes and confidence intervals. Datasets of the study are available at OSF website^1^.

### Ethics Statement

Participants were informed about the entire procedure and signed an informed consent form. They were advised of the fact that the tasks would feature some explicit sexual material. All experimental procedures were in accordance with the Helsinki Declaration and the study was approved by the Ethics committee of the National Institute of Mental Health, Klecany (No 47/16).

## Results

### Modified Dot-Probe Task

**FIGURE 2:**
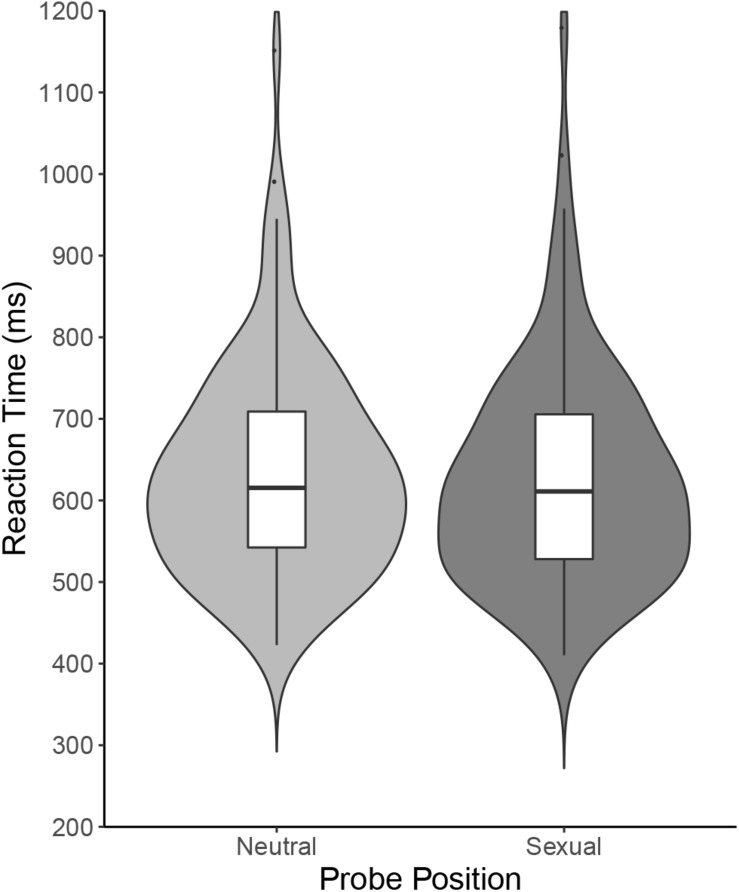
Dot-probe task: Reaction times (ms).

Wilcoxon’s signed-rank test showed that the difference in response times between sex target trials (*Mdn* = 611.06 ms) and neutral target trials (*Mdn* = 615.41 ms) was not statistically significant (W = 3868, *p* = 0.064, *Hodges–Lehmann estimate of Mdn difference* = 4.520 ms, 95% *CI* [–0.247; 9.836], matched rank biserial correlation *r*_*B*_ = 0.201, and 95% *CI* [–0.009; 0.394]). Further, Mann–Whitney *U* test showed that the difference in attention bias index between men (*Mdn* = 2.495 ms) and women (*Mdn* = 2.340 ms) was not statistically significant either (W = 1448, *p* = 0.633, *Hodges–Lehmann estimate of Mdn difference* = –1.083 ms, and 95% *CI* [–6.113; 3.659], matched rank biserial correlation *r*_*B*_ = –0.054, and 95% *CI* [–0.266; 0.163]). See Figure 2 for illustration and Table 1 for a summary.

All reliability estimates were well above 0.80 (see Table 2) and statistically significant (all *p* < 0.001).

**TABLE 2:** Dot-probe task: Split-half reliability estimates.

	**Split half *r***	***CI* 95%**	**ρ_*SP*_**	***p***
All trials	0.894	[0.849; 0.926]	0.944	<0.001
Sex target	0.881	[0.831; 0.917]	0.937	<0.001
Neutral target	0.881	[0.831, 0.917]	0.937	<0.001

*ρ_*SP*_ = split-half reliability coefficient predicted by Spearman–Brown formula.*

### Picture Recognition Task

Mauchly’s test indicated that the assumption of sphericity for the ANOVA was violated in the analysis of hit rates [*χ^2^*(2) = 0.551, *p* < 0.001], which is why degrees of freedom were corrected using Greenhouse–Geisser estimates of sphericity (ε = 0.696).

**FIGURE 3:**
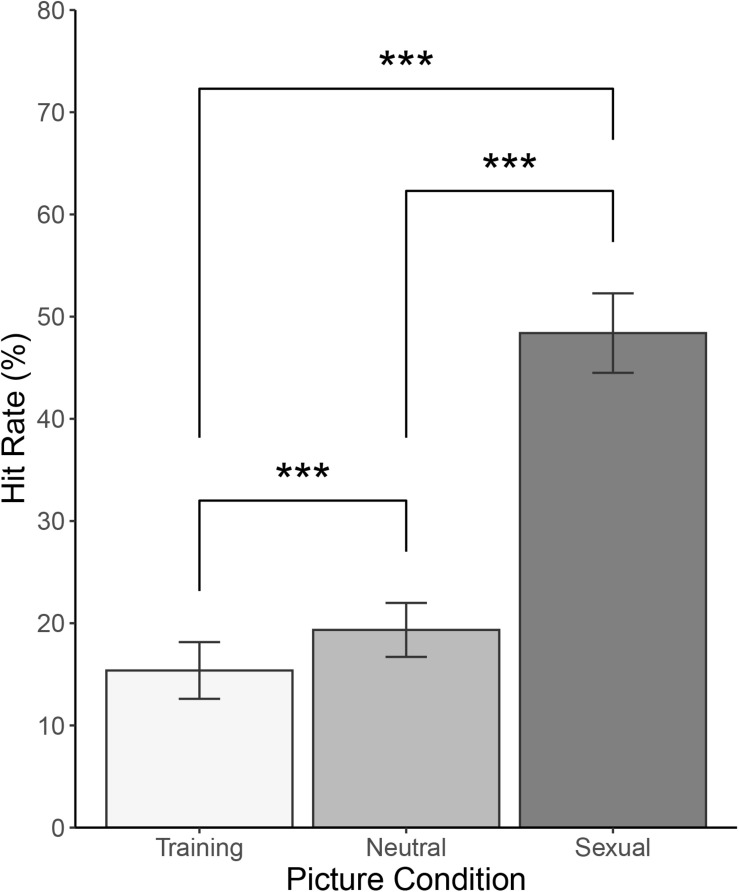
Picture recognition task: Correct recognition (%) for training, neutral, and sexual pictures. ***p < 0.001.

There was a main effect of Category [F(1.380, 153.222) = 213, *p* < 0.001, and *ω^2^* = 0.427]. *Post hoc* tests using Bonferroni correction revealed that sexual pictures’ hit rate (*M* = 48.4%, *SD* = 21.1) was greater than neutral pictures’ hit rate (*M* = 19.3%, *SD* = 14.3, *d* = 1.461, 95% *CI* [1.193; 1.724], and *p* < 0.001), sexual pictures’ hit rate was greater than training pictures’ hit rate (*M* = 15.4%, *SD* = 15.0, *d* = 1.445, 95% *CI* [1.180; 1.708], and *p* < 0.001), and neutral pictures’ hit rate was greater than training pictures’ hit rate (*d* = 0.353, 95% *CI* [0.162; 0.542], and *p* < 0.001). There was no statistically significant main effect of Sex [F(1, 111) = 0.521, *ω^2^* = 0, and *p* = 0.472] or interaction between the two factors [F(1.380, 153.222) = 2.335, *ω^2^* = 0.005, and *p* = 0.118]. See Figure 3 for illustration and Table 3 for a summary.

**TABLE 3:** Picture recognition task: Mean accuracy scores (%), median, and *SD*.

	**All (*N* = 113)**			**Men (*n* = 45)**			**Women (*n* = 68)**		
	**Mean**	**Median**	***SD***	**Mean**	**Median**	***SD***	**Mean**	**Median**	***SD***
Sexual	48.389	48.0	21.091	49.867	50.0	21.786	47.412	45.0	20.724
Neutral	19.345	16.0	14.326	16.356	14.0	13.405	21.324	18.0	16.667
Training	15.376	10.0	15.049	13.556	10.0	14.157	16.581	12.5	15.597

Mauchly’s test indicated that the assumption of sphericity for the ANOVA was violated in analyses of *d’* and *c* [*χ^2^*(2) = 0.731, *p* < 0.001], which is why degrees of freedom were corrected using Greenhouse–Geisser estimates of sphericity (ε = 0.788).

**FIGURE 4:**
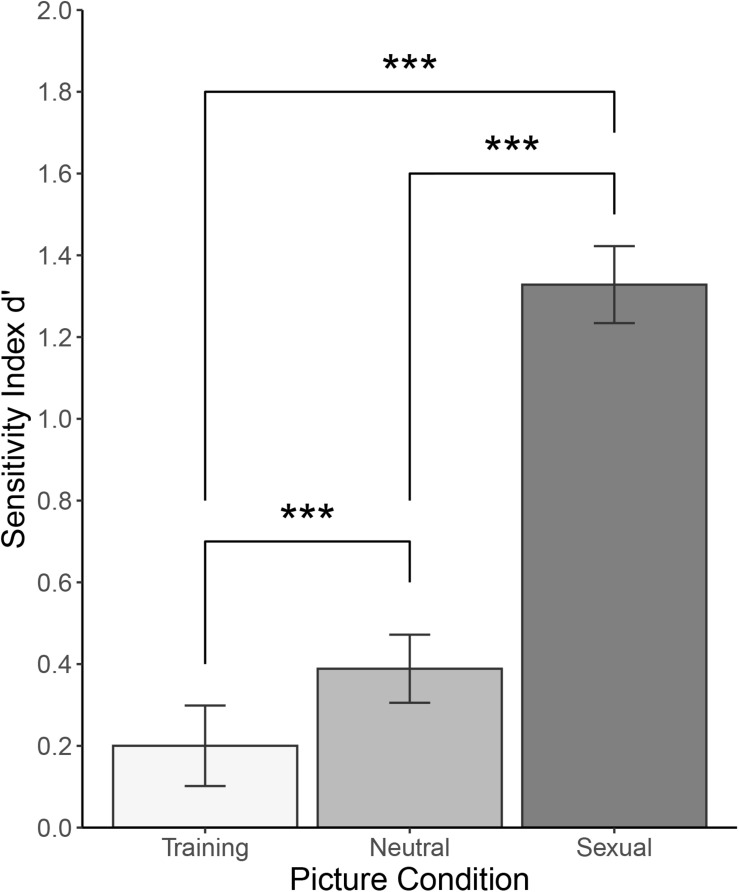
Picture recognition task: Sensitivity index *d’* for training, neutral, and sexual pictures. ***p < 0.001.

For sensitivity index *d’*, there was a main effect of Category [F(1.576, 174.911) = 208, *p* < 0.001, and ω^2^ = 0.494]. *Post hoc* tests using Bonferroni correction revealed that participants showed greater sensitivity to sexual pictures (*M* = 1.328, *SD* = 0.511) than to neutral pictures (*M* = 0.389, *SD* = 0.452, *d* = 1.510, 95% *CI* [1.239; 1.779], and *p* < 0.001), greater sensitivity to sexual pictures than to training pictures (*M* = 0.200, *SD* = 0.534, *d* = 1.480, 95% *CI* [1.211; 1.746], and *p* < 0.001),and greater sensitivity to neutral pictures than to training pictures (*d* = 0.386, 95% *CI* [0.194; 0.576], and *p* < 0.001). There was no statistically significant main effect of Sex [F(1, 111) = 0.070, *ω^2^* = 0, and *p* = 0.792] or interaction between the two factors [F(1.576, 174.911) = 3.305, *ω^2^* = 0.011, and *p* > 0.050]. See Figure 4 for illustration and Table 4 for a summary.

**TABLE 4:** Picture recognition task: Mean sensitivity index *d’*, median, and *SD*.

	**All (*N* = 113)**			**Men (*n* = 45)**			**Women (*n* = 68)**		
	**Mean**	**Median**	***SD***	**Mean**	**Median**	***SD***	**Mean**	**Median**	***SD***
All trials	0.759	0.731	0.358	0.754	0.774	0.408	0.763	0.727	0.324
Sexual	1.328	1.378	0.511	1.402	1.401	0.564	1.279	1.345	0.470
Neutral	0.389	0.429	0.452	0.278	0.317	0.430	0.462	0.455	0.454
Training	0.200	0.265	0.534	0.206	0.311	0.511	0.197	0.225	0.552

**FIGURE 5:**
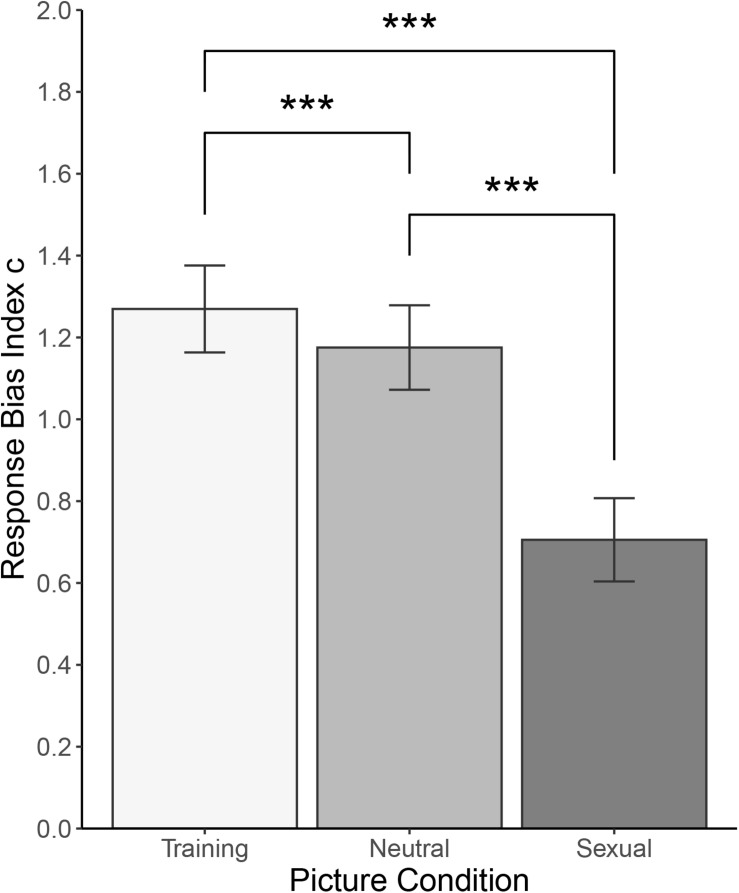
Picture recognition task: Response bias *c* for training, neutral, and sexual pictures. ***p < 0.001.

For response bias index *c*, there was a main effect of Category [F(1.576, 174.911) = 208, *p* < 0.001, and ω^2^ = 0.160]. *Post hoc* tests using Bonferroni correction revealed that participants showed smaller response bias toward sexual pictures (*M* = 0.706, *SD* = 0.552) than toward neutral pictures (*M* = 1.175, *SD* = 0.560, *d* = 1.510, 95% *CI* [1.239; 1.779], and *p* < 0.001), smaller response bias toward sexual than toward training pictures (*M* = 1.270, *SD* = 0.576, *d* = 1.480, 95% *CI* [1.211; 1.746], and *p* < 0.001), and smaller response bias toward neutral than toward training pictures (*d* = 0.386, 95% *CI* [0.194; 0.576], and *p* < 0.001). There was no statistically significant main effect of Sex [F(1, 111) = 0.026, *ω^2^* = 0, and *p* = 0.872] or interaction between the two factors [F(1.576, 174.911) = 3.305, *ω^2^* = 0.002, and *p* > 0.050]. See Figure 5 for illustration and Table 5 for a summary.

**TABLE 5:** Picture recognition task: Mean response bias index *c*, median, and *SD*.

	**All (*N* = 113)**			**Men (*n* = 45)**			**Women (*n* = 68)**		
	**Mean**	**Median**	***SD***	**Mean**	**Median**	***SD***	**Mean**	**Median**	***SD***
All trials	0.990	1.048	0.500	0.997	1.045	0.565	0.985	1.054	0.456
Sexual	0.706	0.765	0.552	0.673	0.683	0.627	0.727	0.798	0.499
Neutral	1.175	1.221	0.560	1.235	1.250	0.631	1.136	1.175	0.508
Training	1.270	1.332	0.576	1.272	1.340	0.646	1.268	1.289	0.530

### Attention Bias and Questionnaires

We found positive and statistically significant correlations between attention bias index and the SOI-R Attitude subscale [*r*_*s*_(100) = 0.274, 95% *CI* [0.084; 0.445], and *p* = 0.005] and between attention bias index and SOI-R Desire subscale (Desire subscale: *r*_*s*_(100) = 0.232, 95% *CI* [0.040; 0.407], and *p* = 0.019). There was no statistically significant correlation between attention bias index and either the SOI–R Behavior subscale (*r*_*s*_(100) = 0.147, 95% *CI* [–0.048; 0.331], and *p* = 0.139) or BDI–II sum score (*r*_*s*_(103) = 0.078, 95% *CI* [–0.116; 0.266], *p* = 0.430). See Table 6 for descriptive statistics.

**TABLE 6:** Questionnaires: Mean, median, and *SD*.

	**Mean**	**Median**	***SD***
SOI	36.216	35.0	14.635
Attitude	16.382	16.5	7.035
Desire	11.621	11.0	5.829
Behavior	8.214	7.0	5.618
BDI	11.848	9.0	9.254

## Discussion

The aim of present study was to find further support for visuospatial attention bias toward sexual stimuli in a nonclinical population as measured by the DPT, to validate its results using the PRT, and to check for sex differences while controlling for depressive symptoms and sociosexuality.

Contrary to all previous research which worked with the dot-probe paradigm for sexual pictures, our main findings show no evidence of bias in favor of sexual pictures in our data. We found a very small difference of 4.520 ms in reaction times between sex target and neutral target trials. Such difference is simply not large enough to reach either statistical or practical significance.

What we did find, however, was a large difference in hit rates for all experimental categories of pictures presented in the PRT. In memory research, exposure time is directly linked to recognition performance (Martini and Maljkovic, 2009). If the DPT works as intended, and if there is an effect to be found, participants should attend mostly to sexual pictures and therefore recognize significantly more pictures from this category. It turned out that participants were indeed most successful in recognizing sexual pictures, achieving a hit rate of almost 50%. One might be tempted to explain this as random responding but in the light of markedly lower hit rates for neutral and training pictures, it is probably not the case. Moreover, because neutral pictures were presented to each participant twice (once in each block), while training pictures were presented only once, one could expect better recognition memory for neutral pictures. Our results showed that neutral pictures were successfully recognized at just below 20% rate and training pictures at 15% rate, which lends further support to the dot-probe paradigm working as intended.

The PRT design did, however, have one limitation that prevents us from making stronger claims based on its results: due to the procedure of stimuli selection for the DPT, there were no clear sexual and non-sexual distractors but rather just a range of more or less easily categorized pictures. This might have affected participants’ response patterns (enabling, for example, a strategy of rating most pictures with sexual content as “seen” in the PRT). For assessing this potential bias, we computed sensitivity index *d’* and response bias index *c*.

Sensitivity in the context of signal detection theory shows a degree of overlap between signal and noise distributions. The larger the index, the larger the participants’ ability to distinguish signal from noise. Once again, though, we found the same pattern as with the hit rates. By far the largest *d’* was found for sexual pictures, much lower value for neutral pictures, and even lower for training pictures. Such results suggest that sexual pictures were indeed more easily remembered and distinguished from distractor pictures.

Response bias is a general tendency in responding and can reveal potential problematic response patterns. Negative values signify a bias toward a yes response (“seen”), while positive values signify bias toward the no response (“not seen”). Our results showed a relatively strong bias toward the no response with the weakest bias for sexual pictures, stronger for neutral pictures, and strongest for the training pictures. While there is no bias toward generally positive responses, this suggests that participants felt more certain about marking a sexual (rather than neutral or training) picture as “seen,” possibly because they recognized those pictures more frequently. The recognition task thus represents a strong, albeit indirect, confirmation of the assumption which the DPT use is based on, namely that sexual pictures indeed attract spatial attention.

It is but natural that one should ask why the present study detected no attention bias toward sexual pictures in the DPT while finding the evidence for memory bias in PRT. There are several possible explanations:

1) *Our results may reflect specific variations in the methodology*. The 500 ms presentation time may have given participants enough time to freely shift attention before the probe appeared (Cooper and Langton, 2006; Jiang and Vartanian, 2018), stimuli may have been too weak to capture attention, or they may have been too complex (Miller and Fillmore, 2010), or perhaps the more demanding probe-identifying task diverted participants’ attention. The main problem with all these explanations is that if small methodology changes weakened the effect as significantly as our results seem to suggest, the effect itself may not be as universal and robust as theorized.
2) *The study may have insufficient power to reliably find the effect*. If that were the case, then given that with sample size of 113 the study had a 90% probability of finding the reported effect had it been of size of at least 0.34, the real effect would have to be much smaller, possibly on the lower bound of reported confidence interval (Strahler et al., 2019).
3) Our findings may be a not so rare case of false negative results.
4) *The dot-probe task may be a poor measurement instrument*. Although we found our results robust and reliable, as indicated by the split-half reliability estimate, the DPT measure as such has been criticized for extremely weak reliability (Schmukle, 2005; Staugaard, 2009). It has also been claimed that the DPT cannot effectively distinguish between attention bias and the SCID and that it struggles with producing reliable findings in other research areas as well (see Kruijt et al., 2019; Strahler et al., 2019). This explanation is partially supported by our PRT results which do suggests attention bias for sexual pictures.
5) *The most obvious explanation is that there is no visuospatial attention bias toward sexual pictures*, at least not in a form that can be measured by the DPT, or that the bias is exceedingly small and easily drowned in a measurement error. Even the evidence from the PRT is somewhat mixed. Sexual stimuli seem to be prioritized in memory, as shown by high free recall rates (Bush and Geer, 2001; Pottage and Schaefer, 2012; Bradley et al., 2017), and a similar effect may well apply to recognition of sexual pictures. In the context of our study, the 48.4% hit rate could be the result of participants attending to sexual stimuli only 50% of the time but having a strong memory bias for them. But to the best of our knowledge, this area has not been explored yet. Other kinds of evidence in favor of existence of attention bias toward sexual images are not quite convincing either. Eye-tracking studies which show early attentional orientation toward sexual pictures (Lykins et al., 2006; Fromberger et al., 2012) were seriously underpowered. The constantly increasing use of erotica in advertisement (Reichert et al., 1999; Reichert and Carpenter, 2003) may be explained by other properties of sexual stimuli, namely its arousing nature and its effect on memory, mentioned just above. To date, there is no convincing evidence for visuospatial attention capture by sexual stimuli (Imhoff et al., 2019; Strahler et al., 2019).

Our sample did not differ in depressive symptoms from normal population, which is why it is most unlikely that depressive symptomatology had any effect whatsoever on the attention bias we measured. On the other hand, we found a weak but significant correlation between attention bias, SOI-R Attitude subscale, and the SOI-R Desire subscale. Higher ratings of openness to uncommitted sex and higher sociosexual desire were linked to a faster identification of probes in sex target trials. These results are clearly divergent from Prause et al. (2008) finding of a negative relationship between attention bias and sexual desire. Even so, confidence intervals indicate that the real effect may be extremely small. We see this inconsistency with previous research as yet another sign of poor reliability of the dot-probe paradigm.

Research on attention bias to emotional stimuli in general and to sexual stimuli in particular has clear theoretical and clinical implications. General (cognitive and visuospatial) attention bias is an important part of theories on sexual cognitive processing (Barlow, 1986; Bailey et al., 1994; de Jong, 2009). Failure to find such biases would indicate a need to revise these theories. But even aside from that, if we were able to establish the general magnitude and direction of visuospatial bias and validate a simple tool to measure, it would help us disentangle and better understand the functioning of attention processing for sexual stimuli. It would also help clinical practice. For example, greater attention bias toward sexual stimuli might signal problems in controlling urges and thus indicate being at risk for impulsive sexual behavior, while lack of attention to sexual stimuli might be linked to low sexual arousability and some sexual disorders (Strahler et al., 2019).

## Conclusion

The present study employed a modified dot-probe task to measure visuospatial attention bias toward sexual pictures in a nonclinical population. Additionally, we used a simple PRT to assess DPT validity. Although the findings of the PRT did suggest increased attention toward sexual pictures, DPT results showed no effect whatsoever. Moreover, we found no differences between men and women. We discussed several implications and possible explanations of our null result. In conclusion, both our findings and the literature we reviewed suggest that attention bias toward sexual pictures is either rather small, that it does not exist in the theorized form, or that the dot-probe paradigm is not a reliable tool to assess it.

## Data Availability Statement

The datasets presented in this study can be found in online repositories. The names of the repository/repositories and accession number(s) can be found below: https://osf.io/3fdtn/.

## Ethics Statement

The studies involving human participants were reviewed and approved by the ethics committee of the National Institute of Mental Health, Klecany (No 47/16). The patients/participants provided their written informed consent to participate in this study.

## Author Contributions

ON: conceptualization, data curation, formal analysis, investigation, methodology, project administration, software, visualization, and writing – original draft preparation. KB: conceptualization, funding acquisition, investigation, resources, validation, visualization, and writing – review and editing. VV: investigation and resources. KK: conceptualization, funding acquisition, methodology, resources, supervision, validation, and writing – review and editing. All authors contributed to the article and approved the submitted version.

## Conflict of Interest

The authors declare that the research was conducted in the absence of any commercial or financial relationships that could be construed as a potential conflict of interest.

## Footnotes

1 https://osf.io/ba5w4/
